# 

**Published:** 1911-02-18

**Authors:** 


### CONTENTS.

| | PAGE |
|---|---|
| **Irish Books and English Reviewers** | 597 |
| **Infantile Mortality and Amateur Hygienists** | 598 |
| **Annotations—** | |
| Monument to Van Beneden — The First Female Doctor of Athens—A Functionary of Napoleon III.—The Encyclopædia Britannica | 599 |
| **Medical Opinion and Movement** | 600 |
| **Hospital Clinics—** | |
| What is Meant by Insanity. By Maurice Craig, M.D., F.R.C.P. | 603 |
| **Laryngology and Rhinology—** | |
| Transillumination of the Nasal Accessory Cavities | 606 |
| **"The Hospital" Medical Book Supplement—No. XXXIX.** | 607 |
| **News and Events of the Week** | 611 |
| **Editor's Letter-Box—** | |
| Pit Ponies | 612 |
| **The Week's Work—** | 613 |
| **Gifts and Bequests** | 14 |
| **Special Institutional Articles—** | |
| The Lighting of Hospitals | 15 |
| An American Cottage Hospital | 16 |
| **Parliament and Publications** | 617 |
| **The Week in the Courts** | 618 |
| **Buildings Contemplated and in Progress** | 618 |
| **Institutional Notes and News** | 619 |
| **The Hospital World** | 620 |
| **Jottings** | 621 |
| **Institutional Letter-Box** | 621 |
| **New Appliances and Things Medical** | 622 |
| **The Week's Novelties** | 622 |

